# Between the witness and the observer: what ethnography can learn from James Baldwin

**DOI:** 10.3389/fsoc.2023.1158520

**Published:** 2023-08-03

**Authors:** Jelani I. Ince

**Affiliations:** Department of Sociology, University of Washington, Seattle, WA, United States

**Keywords:** ethnography, racism, reflexivity, method, Baldwin

## Abstract

What is the role of the ethnographer during a time of increased racial hostility, political mobilization to keep racial minorities “in their place,” and commitments to revisionist interpretations of the country's past and projected future? While the traditional, classic ethnographic approach would recommend that the researcher should avoid taking a stance on so-called political matters and merely *observe* them, I argue that that position is insufficient to address the issues that people are currently facing. Ethnography can, and should, do more. Therefore, this essay argues that the role of the ethnographer should be oriented toward what the late author James Baldwin calls *the witness*. The witness is different from the observer because it rejects a positivistic orientation toward ethnographic fieldwork that prioritizes spectatorship to remain “scientific.” To be a witness is to transgress traditional epistemological understandings of ethnography that ignores how the researcher's position within the racial system shapes how one knows and does not know, what one sees and does not see, and how one imagines freedom and justice. Ethnographers can learn from Baldwin's method because it provides a rich vocabulary to describe the inequality that research participants encounter while in the field and embraces the possibility of an apocalyptic future, which is a future that is not guaranteed if we continue to seek neutrality. In this article, I detail three lessons that we can learn from Baldwin's method and status position as the witness: (1) Connecting empire to the global racial order via the international outsider; (2) Paying one's dues as a within-nation outsider; and (3) Representing the wretched as a within-community outsider. These lessons are instructive for ethnographers because they provide a lens to understand classic ethnographies of the past, while not wallowing in the doldrums of present arrangements, and challenges future research to ground reality as it is rather than what it “should” be.

“You are bearing witness, helplessly, to something in which everybody knows. And nobody wants to face.” -James Baldwin (“The Artist's Struggle for Integrity,” a speech given at the New York City's Community Church, 1962).

## Introduction

In the winter of 1971, Nikki Giovanni and James Baldwin met in a London studio to discuss the different approaches of their respective generations to the Black struggle for justice in the United States. The near 2-h conversation for a public television show called *Soul!* was rich with insights about race, gender, sexuality, family, work, and identity– and the generational responses to each of these conditions. The first question that Giovanni asked Baldwin was: why did you move to Europe? Baldwin paused, elicited a classic grin, and provided several explanations. He described the difficulty he had with focusing on the craft of becoming a Black writer while living in the United States and that living in Europe provided him an appropriate distance to grow as a writer.

He also mentioned a pivotal moment: learning about the murder of Martin Luther King, Jr. while living in the south of France. He ended his answer with the following statement (Nikki Giovanni James Baldwin in conversation on ‘SOUL.!”', 2022):

There was no way ever to leave America. I would be a fool to think that there was some place I could go where I wouldn't carry myself with me. Or if there was some way for me to live if I pretended I didn't have the responsibility which in fact I do have. I'm a cat trying to make it in the world because I'm condemned to live in the world.

Baldwin's response is an apt reminder of the importance of leaving what is familiar in order to better understand oneself and one's relationship to one's country. Living in France, quite literally, saved his life. This move allowed him to make sense of his own life and what was happening in the United States during the civil rights movement. The year after Baldwin's conversation with Giovanni, he published *No Name in the Street* (hereafter *No Name*), which is colloquially known as the sequel to his book *The Fire Next Time* (*Fire*). However, the tone in *No Name* was markedly different from his earlier work. Baldwin expressed a deep cynicism– indeed, condemnation– toward his white countrymen, and the American experiment. He forsook the ideas that he presented in *Fire*: that the United States would be able to deliver on the promises embedded within the country's founding documents. Instead, in *No Name*, he turned his gaze toward an international framework that departed from individual solutions to systemic problems. The tone was so piercing that one reviewer asked how “one of the most sensitive writers in the Western world… [could] come to this?” (Ford 102 as quoted in Sinykin, 2020).

It might seem odd to include a discussion of Baldwin in an issue dedicated to sociologists and ethnographers in particular. Baldwin's texts are not included in sociology training programs; it would be a surprise to find his work in courses on sociological theory, method, and even race relations. Nonetheless, the lessons he provided in *No Name* are instructive for ethnographers and the discipline of sociology because, like many scholars who are marginalized, he is not unfamiliar with the pangs of epistemological erasure and the consequences of being misread by the predominately white field of knowledge production. In this essay I will incorporate his insights from *No Name* to show how his work is sociological and that it aligns with other ways of producing knowledge for qualitative researchers. Specifically, I introduce the concept of the witness as a tool that fieldworkers can use while conducting their fieldwork.

Before moving forward, it is important for me to clarify the defining features of the witness. By using the term witness, I am not referring to merely observing phenomena individually, but, more importantly, observing with a pious relationship to history, that is, using one's status position to publicly unveil the hypocrisy of blind reverence to the nation's history and its institutional arrangements. A key argument that I am making is that inhabiting a pious relationship to history is a tool that ethnographers can bring with them as they conduct their fieldwork and write their analyses (Reyes, 2020). Let me discuss an example that might resonate with readers who are familiar with the sociological tradition. Consider the following statement that Du Bois (2014) provides the reader in his preface titled “To the Reader” in *Black Reconstruction*:

It would be only fair to the reader to say frankly in advance that the attitude of any person toward this story will be distinctly influenced by his theories of the Negro race. If he believes that the Negro in America and in general is an average and ordinary human being, who under given environment develops like other human beings, then he will read this story and judge it by the facts adduced. If, however, he regards the Negro as a distinctly inferior creation, who can never successfully take part in modern civilization and whose emancipation and enfranchisement were gestures against nature, then he will need something more than the sort of facts that I have set down. But this latter person, I am not trying to convince. I am simply pointing out these two points of view, so obvious to Americans, and then without further ado, I am assuming the truth of the first. In fine, I am going to tell this story as though Negroes were ordinary human beings, realizing that this attitude will from the first seriously curtail my audience.

Du Bois had to be explicit about the importance of dispelling the lies that many believed about Black people. But, he makes it clear that he is not writing for readers who believe that Black people are inferior, and that this inferiority is a social, observable fact.^1^ In the preface, he does not express reverence for the Civil War or frame slave masters and their allies in the chattel slavery economy as victims of their time whose agency was swallowed up in this economy of social death (Patterson, 1982). Instead, he states that he is writing to honor Black people's humanity. This is not just a rhetorical strategy—this is the execution of a research strategy and an instance of what it means to inhabit the role of the witness.^2^ One of the most important decisions a researcher makes is where they begin their analysis, which includes shifting the reader's attention to privilege the narratives that center the humanity of marginalized groups to tell the full story of inequality.

Like Du Bois, Baldwin's writing is prolific because he brings what West (1989) labels “prophetic pragmatism” to understanding social phenomena and the world(s) we inhabit. Throughout his career, Baldwin practiced an intense public intellectualism to remind his countrymen of the routes/roots that have been taken to arrive at this particular cultural moment. In the 1960s and 1970s he forwarded a discourse on Black American citizenship and American democracy to disrupt the traditional American narrative that our arrival here is coincidental or accidental. The pious relationship to history must serve to understand the structures of oppression of the present and imagine and work toward building new arrangements in the future. In this way, he took on the burden of the public witness to propose (re)building new arrangements in lieu of the ones we currently have.

To inhabit the role of the witness entails: (1) reflecting deeply about one's own history and place in the world (one's family, social relationships, and position within the nation's institutions), so that (2) one can do the collective work of participating in the ongoing discourse of “fresh understanding [and] greater resolve” to pursue freedom for all people (Benjamin, 2022). Baldwin sees collective freedom and personal responsibility as inseparable. Indeed, his own definition of freedom required acknowledging personal responsibility, an “honest appraisal of the historical roots, as well as the current conditions, of one's situation” (Balfour 131, as cited in Muyumba, 2014). The witness understands personal responsibility not as an individual accomplishment. Instead, the purpose of acknowledging personal responsibility is as Muyumba (2014, p. 164) describes it: “as a set of contingent practices: *phronesis*– active sagacity, thoughtful action; intellectual experimentation and invention; and rhetoric” so that one can “create exchange and participation among the members of a public community, thus inspiring them to make their individual, personal ideas into ***shared concerns and solutions***” (author emphasis).

This is something that Baldwin contributes not only as a writing style, but also as a contribution for ethnography. With his witnessing of the Black American condition, Baldwin made Black American issues, which would otherwise be considered a private, individual concern, a shared, public concern that was inseparable from the nation and its existence. The fate of the country and, importantly, the project of democracy was impossible to determine without also acknowledging Black humanity, suffering, and struggles for complete citizenship.

Drawing on the experiences of the oppressed as a starting point for research and knowledge production is critical for his assessment of the country, his countrymen, and any hope (if at all) for change. In addition to centering the voices of the oppressed, Baldwin adds another element that is important for ethnographers to remember: you need to make the connections between what you observe in the service of building a collective knowledge that produces sociopolitical changes. Ethnographers who are interested in doing the work of the witness must produce knowledge in the service of pushing democracy toward radical change. Our work must be created for a public that is concerned with mutual human recognition as a critical practice.

To be sure, knowledge production cannot happen accurately without taking account of who you are and where you are in systems of oppression, ranging from your biases and prejudices to the training program where you received your methods training (Reyes, 2022). However, witnessing is more than just about participating in personal reflexivity. There are a number of scholars that have already thought deeply about reflexivity (see Davies, 2008; Lichterman, 2017), but the witness pushes us to go further than that. The witness takes on the task of advocating for a radical change in the country's social institutions. Ethnographers who are interested in this type of work do not only need to take responsibility for the harm we might cause in the service of knowledge production, but also what we stand to lose if we do not relinquish the status quo and the fantasies that protect it.

In order to locate the mechanics of this witnessing work and how it aligns with the oeuvre of ethnographic research that wrestles with the researcher's role in the field, this essay considers the following questions: (1) What techniques does Baldwin employ to identify the responsibilities of witnessing?; (2) Which grounds of witnessing are especially important to him? and, (3) Can anyone be a witness? My implicit argument is that paying close attention to Baldwin's engagement with the imperial orders in the United States and France is important to understand his scholarly– in this case, sociological– self, and his literary self. Baldwin's (re)presentations of the United States and France in *No Name* reflect and magnify the tensions between multiple layers of “outsideness”: being an international outsider, a within-nation outsider, and a within-community outsider.

Comparing his observations and experiences in the United States and abroad is useful for ethnographic research because witnessing the interactions between the French and Algerian people, between white and Black people living in the South, and between members of the Black Panther Party demonstrate that reorienting the center to focus on marginalized groups does not “taint” the production of science. My interest lies less with privileging one system of representation over the other (i.e., ditching the “old,” positivist ethnography for the “new,” critical ethnography). Although those debates are still happening, I am more concerned with extending the genealogy of writing and thinking about racism, as well as traditional understandings of racism, racialization, and how that matters for life outcomes. Ethnography is constantly subject to criticism because of claims that it lacks generalizability, yet it is an effective, powerful, and accessible research method that can explain how and why social problems are so difficult to eradicate. It is especially needed right now.

Indeed, ethnographies are socially conceived products that emerge at particular historical junctures and are formed through a dense constellation of complementary and competing bodies of knowledge. Very often, the distinction between these bodies of knowledge represents different chapters in the story of this method, including using the method for the purposes of exploitation and colonial expansion in the service of forwarding a global color line (Itzigsohn and Brown, 2020; Johnson, 2020). Indeed, crucial to overturning the normalcy and neutrality of racist framings of people's behavior (very often, but not always, conducted by white scholars) are the stories by people of color whose experiential knowledge of structural racism provides the “necessary contextual contours to the seeming ‘objectivity' of positivist perspectives” (Ladson-Billings, 1998, p. 11). In that spirit, I am writing this essay to ethnographers writing, researching, and acting in good faith, who are looking for accessible ways to make sure that they do not produce harm with their work and who have different responsibilities to communities outside of the scientific community.

I present the concept of the witness to not only honor the tradition of the critical work that preceded me, but to also show current and future ethnographers how this position can be a part of their “strategic toolkit” while in the field and representing the data they find from their projects (Reyes, 2020). I argue that the position of the detached, neutral observer is insufficient to address the issues that people are currently facing. I make this argument to touch on a more important part of the ethnographic method: responsibility for pursuing freedom for all people, rather than merely describing the conditions of their circumstances. When it pertains to the study of racism one cannot fully grasp all of the dimensions of the observed problem by merely observing the horrors of the worlds we inhabit from a distance. Indeed, it is difficult to argue that the world needs to be changed, but then tow the line of moral non-involvement for the sake of science. By moral non-involvement, I mean juking the responsibility that you have, in private and public, to change and redefine yourself and using that knowledge and experience so that others might live.

In what follows, I will briefly discuss several intellectual interlocutors that speak to the theme of witnessing, especially Black feminists and transnational and postcolonial feminists. I will discuss how these approaches align with, and depart from, other forms of ethnographic practice within sociology. Then, I will turn our attention to the concept of the witness and how it is useful for ethnographers by conducting an exegesis of *No Name*. Specifically, I will discuss three important witnessing “moments”: his observations of the French/Algeria conflict, his observations of Black life in the South in the building civil rights movement, and his relationship with the Black radical tradition via the Black Panthers. As this essay will detail, part of what ethnographic research can tell us is why people commit so heartily to belief systems and practices that not only harm others, but also themselves. Baldwin reminds us that racism endures because of the lies that people delude themselves into believing.

## The legacy of witnessing in ethnographic research

I am not the first person to propose that qualitative, and especially ethnographic, research should be conducted differently. There are many branches of qualitative research that equip the researcher to consider multiple vantage points to understand social problems, such as participatory-action research (McIntyre, 2007) and Black feminist (Collins, 1990, 2000, 2009; Crenshaw, 1991) perspectives. I will pay particular attention to the work of Black feminist and transnational scholars in this essay. An important tenet of the Black feminist tradition is that it is an intellectual tradition that honors previous iterations and theories. It builds upon itself, across time, in order to locate and piece together a language that can accurately represent the experiences, joys, struggles, and power of Black women (Luna and Laster Pirtle, 2022). Black feminism is important despite the attempts to suppress, ignore, and erase the contributions of Black feminist scholars. For instance, Collins (2000, p. 8) lists several examples of suppression, but the one that matters especially for this essay is “paying lip service to the need for diversity, but changing little about one's own practice.” By this, Collins draws our attention to how scholars talk about acknowledging the importance of diversity, but making little change to their citation practices and their paradigms to understand today's problems.

In this way, witnessing is part of that paradigm shift that scholars need to do. To take on the task of witnessing involves centering the epistemological insights from those who occupy the status position of the “outsider-within” (Collins, 1986). Individuals who live at the intersection of multiple systems of oppression can use that position to not only resist inequalities of power, but to better understand and observe these systems of domination. Race, class, and gender scholars have long argued that the margin is a place of oppression and resistance from where marginalized groups can cultivate reflexive perspectives (Du Bois, 1903; Fanon, 1961; Rawick, 1972; Collins, 1986, 2000; Crenshaw, 1991). As bell hooks (1984) reminds us, the margin can enable subordinated people to look “both from the outside in and from the inside out,” which helps develop a “second sight” (Du Bois, 1903: p. vii), a “mode of seeing unknown to the oppressors.” Indeed, being in an outsider-within position can be advantageous, as it enables people to make “creative use of their marginality” (Collins, 1986, p. 14).

It is possible to draw from the lived experience to make sense of the observations, interactions, and “moments” that people encounter, to create theory. One does not, and should not, need to be a distant observer to lay claim to knowledge production. Therefore, to occupy the position of the outsider-within opens new pathways to consider how we demonstrate what we observe and what we can know, with authority. In other words, witnessing can be included within the oeuvre of Black feminist thought. Too often, the voices of Black, feminist, and critical scholars are suppressed, ignored, and dismissed because they depart from the traditional way of doing science. Witnessing from the perspective of Black people opens the possibility to build coalitions that support Black freedom and therefore *everyone's* freedom. It is imperative to reject the short-sighted, zero-sum game that argues that prioritizing Black people's freedom comes at the expense of everyone else's freedom.

The work of transnational and queer feminists is also relevant for discussions of what witnessing can look like, especially when it pertains to troubling and harmful social issues (for both the researcher and the participants). Moussawi's (2021) work examines “bad feelings” in the ethnographic process. Moussawi discusses how to make sense of fieldwork when the researcher encounters negative feelings that uncover past lived trauma, and how the researcher should move forward when their research causes them to feel “bad.” In her essay, Moussawi argues that bad feelings about one's research is stigmatized in the academy and feeling bad about research is a response to the rigid boundaries placed around knowledge production. Emotions are framed as illogical, or antithetical to reason; therefore, this serves to marginalize “certain topics, modes of knowing, and scholarship” (79). This is important for witnessing because transparency about troubling encounters in the field and the impact that has on the researcher is a necessary consideration for the execution of a project. It is important to be mindful of what our limitations are and how this will affect how we conduct our work. It is better to be an honest witness than to ignore one's limitations and potentially misrepresent the people we encounter and the observations we make.

This mindfulness is what Baldwin means when he charges us to consider our personal responsibility in the work. Moussawi is a public witness to a troubling and traumatic subject area; for her, it is not just a matter of executing observations, but taking responsibility for how the work affects her. For Moussawi, the goal is not to produce science for the sake of the academic community, but to do it in a way that does not erase her lived experiences and feelings. Boundaries are important to honor and one's safety should not be sacrificed to conduct research. As other scholars remind us (Parreñas, 2021; Hoang, 2022), fieldwork is an embodied experience and different ethnographers have to navigate different challenges (either personal experience or hearing others encountering it) such as sexual harassment (Parreñas, 2021; Hoang, 2022), or threats to one's bodily capital (Hoang, 2022).

Researchers must, to borrow from Reyes's (2020) conceptualization of “the ethnographic toolkit,” recognize “how both our theoretical traditions and methodological choices are strategically used throughout research” (p. 235). What this means is that researchers who take on the role of the witness must see research participants as humans, not just a means to accomplish an end for the scientific community. For example, in his ethnography of Black youth rappers in South Central Los Angeles, Lee (2016) argues against the use of concepts like “participants,” “research subjects,” and “ideas about in-groups and out-groups” because these terms are inadequate for capturing kinship and other processes of intersubjectivity in qualitative research. These are antiquated terms and ideas that do a disservice to the people who we witness building and changing the social world. The role of the witness builds on these critiques of detaching ourselves to uphold the project of science. The witness can be considered a role to inhabit to make sense of the experiences in one's country (or other sites of field research), interacting with its inhabitants, and connecting those experiences to the broader, global racialized social system. Again, to return to Reyes (2020, p. 25), because “we have multiple characteristics we draw on and we do not share all of our participants' characteristics” our methods should reflect those changes, boundaries, and points of intersection.

## What is the witness, exactly?

In *No Name*, Baldwin considers the building confrontation between the emerging energy of the Black freedom struggle (beginning with the civil rights movement and ending with the Black Panthers) and what Ray and Seamster (2016) have termed the “racial progress narrative” about the country and its liberal views of itself. Baldwin takes issue with the delusion that so many white people continue to believe about the country and its progress, and he does hold back in his criticism of this behavior. You might wonder what distinguishes the witness from a scholar-activist or participant-observer. The witness is not a different “type” of scholar, or a different methodological approach; rather, it is an approach to ethnography that is based on a political commitment to pursue freedom for oppressed communities. It is less about clear criteria that demarcate between these different “types” of scholars. Instead, the witness must consider the motivations for entering the field in the first place, and what criticisms she wishes to forward in the interest of building a new world. Thus, while a participant-observer or scholar-activist might enter the field based on their own research interests, because they locate a gap in a research program, or are driven by a desire to see justice in the world, the witness enters the field on behalf of the interests of the people they are in community with, with a commitment to not sensationalize people's experiences, and to use the lessons learned from experiencing the social to make criticisms about the country's arrangements. I do not think Baldwin would refer to himself as a scholar-activist or bother with labels in this capacity. Baldwin was constantly careful with how he described himself. In the conversation between him and Giovanni that was referenced at the beginning of this essay, both joked about if there was even such a thing as a “Black writer.” He expressed hesitation toward words that are often used to describe writers, too, such as “artist,” “integrity,” and “courage” and instead was more interested in the meaning and reality *behind* those words.^3^

Baldwin wanted to use his writing to challenge people to live courageously and pursue freedom. His work reminds us that he identified as a Black man who wanted to speak truthfully about the horrors that his country refused to accept and continued to propagate. Therefore, I do not think that he would equate witnessing with operating as a scholar-activist or a different type of scholar. To be an effective witness does not require identifying with a particular research camp or tradition, nor does it require one to propose immediate solutions to the inequality that one encounters as a field researcher. Often, and especially within the sociological tradition, research studies end with suggestions to change policies or other formal rules to ameliorate the problem(s) of interest. I am not saying that suggesting policy changes are inherently wrong or misguided; rather, it should not be the only orientation that researchers should privilege when confronted with the uncomfortable weight of our research findings. It should not be the only recommend end for research.

In this regard, the witness has a different relationship to history, knowledge production, and the current arrangements that continue to erase the capacity for human thriving. Specifically, the witness resists the urge to frame inequality under the guise of “pervasive presentism,” where the researcher “conveys an image of the social world as being governed by unchanging universal laws and logics of necessity […] [and] the message is that the present is the same as the past, or that the past is simply not interesting” (Steinmetz, 2018 as quoted in Patterson, 2019). The witness understands that the social world cannot solely be described in the “sociological present tense” (Steinmetz, 2018). To speak honestly about the durée of white supremacy requires utilizing the contributions from different disciplines, theorists, artists, and other creatives who are concerned with the weight of the social.

To inhabit the role of the witness is to shift one's relationship to the project of science. By this, I mean that the witness is not so much concerned with “science” for the sake of protecting its supposed sacrality, but using scientific methods– in this case, observations– for the sake of humanizing people that live and survive within oppressive systems. In *No Name*, Baldwin (1972) refers to himself as a “public witness to the situation of Black people.” His words entail a certain form of responsibility that recognizes the duty to represent “the voice” of a diverse community in the public– and using that platform to criticize any claim to the narratives about the country that suggest its hands are pure, clean, and without fault. At the time that Baldwin wrote this book he was known, nationally and internationally, as an esteemed writer and critic of American race relations. Producers in Hollywood asked him to write a screenplay for a film about Malcolm X, and later a similar film in the wake of Martin Luther King's murder. He declined both offers because he did not want to be an accomplice in “a second assassination” of these men (50). This refusal is important. His refusal tells us that not all representation of your community members, especially those with notoriety, can be considered an honor.

It might be tempting to argue that Baldwin was merely overreacting and that the Hollywood producers did not intend to offend him or, importantly, Malcolm's legacy. Sociologists are trained to avoid arguing about intent because to do so would be considered a moral arrangement, and locating intent would mean that we would have to confront different explanations for why, in this case, the “race problem” mattered for that particular social situation. Nonetheless, this has not prevented scholars from trying to argue that intentions matter in the day-to-day experience in the United States. We have already seen the damage of assuming intentions about the “faces at the bottom of the well” (Bell, 1992). One need only to read the Moynihan Report and subsequent articles that discussed the functionalist “culture of poverty” thesis that continues to plague our political economy (Small et al., 2010). The point here, however, is that regardless of intent, you cannot evade responsibility for your actions. You *are* responsible for the words you produce and the potential responses that the audience of these words will conjure upon interacting with your words.

Baldwin understood this tension, especially as he grew in fame and popularity as a writer in the American context. Nonetheless, this change in status did not protect him from confronting the deeply unsettling ways that his country and countrymen continued to choose their own destruction, and therefore ensure the marginalization of his community. It is here where *No Name* takes a different, more apocalyptic tone, than his previous texts, such as *Fire*. In the latter text, Baldwin expresses hope for change and belief that his country can turn a corner toward progress. However, ten years later, he provides a different, more refined argument about the durée of the racial order in the United States and, as we will see soon, the rest of the world. Here, we turn our attention to the first lesson: how witnessing as an international outsider bears lessons for connecting the domestic racial order to a global racial order.

## Lesson 1: connecting empire to the global racial order via the international outsider

Before Baldwin arrives in France, there was already a decolonial project underway in the country. The Vietnamese are fighting against French occupation in the First Indochina War (1946–1954). This led to a standoff at Dien Ben Phu in 1954, where the Viet Minh overwhelmed French forces and force the French to retire their efforts to maintain power over what was then known as colonial French Vietnam. Almost immediately, the French become entangled in the Algerian War (1954–1962). Baldwin's original reason to retreat to Europe was to escape the vice grip of antiblackness in the United States. He is convinced that the country and its social institutions will kill him, or that he will kill someone. Yet, he notices a deep hostility toward Algerians in France is well underway. He begins to make sense of his position as an American citizen and Black man within this tapestry of colonial relations between France and Algeria. He is *not* an insider or member of either community. In fact, he arrives in France penniless, a stark status shift from his experience in the United States. There is no limo to pick him up from the airport and take him to wherever he wants to go in the city. Consequently, he finds himself living “mainly among les misérables, and in Paris, les misérables are Algerian” (56). Indeed, he finds a community in sharing status with those whose faces were also at the bottom of the well (Bell, 1992). Because he lived with les misérables, or in Fanonian terms “the wretched,” he observes an incredibly rich life of interactions between Algerians living in France, and Algerian/French citizen interactions to make sense of his own marginalization, and position, as a Black American (Fanon, 1961).

As a result, he sympathizes with the plight of the Algerian community because they are treated similarly to his community in the United States. His observations of the Algerian condition in France leads him to conclude that when empires are threatened and confronted, the response is one of greater force, as it can no longer pretend to justify itself. In fact, the first time he uses the term “witness” in the text is when he observes the French police exert violence toward an unassuming Algerian man by throwing him through a glass door and leaving him in the street. He writes (Baldwin, 1972) that he “witnessed a murder, or nearly witnessed an attempt to do that.” According to his observations, the French were hurt that “their stewardship should be questioned, especially by those they ruled.” Even though the Algerians had nothing to do with the defeat in Vietnam, Baldwin notices that the French police viewed Algerians as a threat to their authority and, largely, the authority of the French empire. He not only observed this through interpersonal interaction. He was not content with merely observing the violence in the country to which he was an outsider. He also pairs his observations with content analysis of *Combat*, a journal run by Albert Camus to see how Algerians are framed in French media. He makes his observations of the French/Algeria conflict not for the sake of scientific ambitions, but to make sense of the *global* racialized order that all of us are a part of. His study of these events led him to a larger point: colonialism is not just present in France, but it affects all non-white people around the world.

Here is the lesson that is instructive for ethnographers: through his observations of social interactions and content analysis he collapses the demarcation between “comparison groups” across the colonial color line and instead gathers empirical data from the vantage point of socially marginalized groups. Indeed, there is enough explanatory power about the dimensions of inequality that can be observed within a socially marginalized group, instead of assuming homogeneity within this group or that the outcomes of this group need to be compared to the experiences of members from dominant groups. He utilizes his observations to leverage the act of witnessing toward social justice. He walks with the Algerian people, lives with them, eats with them– this labor of participation provides him a language to reflect the Algerian people's physical, social psychological, and cultural condition. Notice, though, that this is a slight departure from participatory-action research. Baldwin does not collaborate with the Algerian people to “develop research questions, methodologies, and analyses that empower and liberate communities” (Hayes et al., 2022, p. 102).

Nonetheless, his observations challenge the audience to outline the afterlives of colonialism and the ever-present need for empires to assert their dominance over countries and people they have determined to be inferior. Baldwin does not include interviews with French police officers or citizens to get “both sides” of this conflict. Indeed, “the Algerians were not fighting the French for justice [...] but for the power to determine their own destinies” (Baldwin, 1972). His status as a witness is not to pursue distant objectivity, but to make a moral commitment toward honoring the Algerian people as theorists of their own condition, rather than viewing them through the eyes of the dominant class. The longer Baldwin stays in France, the more connections he makes to his own experiences as a Black man in the United States. He goes on to write that it is “strange to find oneself, in another language, in another country, listening to the same old song and hearing oneself condemned in the same old way” (Baldwin, 1972). This reflection draws a comparison between French colonialism and the vestiges of institutional and systemic racism in the United States. At the same time, he is also deeply aware of how living in France isolates him from the very people (and problems) he wants to speak to, connect with, and protect: Black Americans. This desire to be in community back home, in part, allows him to see how insulated he was while witnessing the Algerian/French conflict because what was happening to them “did not appear to be happening to the blacks” (Baldwin, 1972).

While he could understand and support the Algerian independence project, he could not fully detach himself from the United States cultural frame. He concluded that he was, “operating, unconsciously, within the American framework, and, in that framework, since Arabs are paler than blacks, it is the blacks who would have suffered most” (Baldwin, 1972). He believes that he needed to return back to the United States. In his own words: “Everybody else was paying their dues, and it was time I went home and paid mine” (Baldwin, 1972). Now, we will turn our attention toward how his responsibility as a public witness manifested after his return to the United States, specifically as a within-nation outsider.

## Lesson 2: paying one's dues via the within-nation outsider

Baldwin returns to the United States in 1957 in the midst of the growing civil rights movement. He misses the familiarity of the country– the sounds of taxis in New York City, the familiar places, his family, and, yes, even the food. However, underneath this nostalgia is a recognition that his work is unfinished in the United States. While in Paris, he operated as a witness for the Algerian condition in the French colonial regime. Now, he must turn back to his own country to engage in the unfolding institutional and systemic racism of his birthplace. This colonial comparative analysis profoundly reshaped Baldwin's prose. Upon returning to the United States, he changes his language; Baldwin begins to frame Black Americans as a colonized people, and white people as colonizers. This language shift is influenced, no doubt, by witnessing the surveillance and subordination of the colonial regime in France.

For Baldwin, paying one's dues is an important burden to carry if one is to conduct the work of the witness. To pay one's dues involves developing deep, interpersonal intimacy with the community/communities that you are walking alongside and taking careful attention to describe one's people in humane (rather than scientific) terms (Du Bois, 1903). By humane terms, I mean describing Black people as complex, beautiful, and fiercely determined people, rather than hapless victims, unintelligent recipients of white supremacy, or individuals who are “cultural dopes” (Lynch, 2007). Instead, Baldwin's witness aligns with what the late Bell (1991, 1992) refers to as racial realism– a mindset or ideology that describes how Black people navigate the terrain of unrelenting white supremacy on the interpersonal, institutional, and systemic levels, and sustain themselves in the midst of this reality. The racial realist accepts the permanence of racism as a necessary staple within the United States, but does not wallow in this reality; instead, the realist seeks (and imagines) alternative avenues of being that fall outside of the periphery of the status position that the country has relegated her to. The racial realism approach is useful here because it provides a broader articulation of the Black experience that exceeds white sociological framings of Black life– as colorless, or merely a site of permanent exploitation. With this turn in *No Name* Baldwin's observations help us understand the colorful moments of “Black placemaking” (Hunter et al., 2016). This is an aspect of witnessing that is important to him as he turns his attention to the South in particular.

Baldwin is commissioned to write a newspaper article on how Black people are treated in the South. The assumption was that Baldwin would write about the “typical” Black experience under Jim Crow and that this work would help solidify him as a voice in the movement. In contrast, Baldwin approaches this writing project with the understanding that the troubles that face the South are not unique to that region. The origin of the so-called “Negro problem” is both an international problem manufactured by the global racial order *and one* that emerges from within private life. In the U.S. South, for example, Black people have long been subject to the whims of white people who think about them as “mammies,” “magical Negroes,” and as hyper-sexual beings– all of which shapes Black bodies and lives into beings that can be controlled in all arenas of the social. To this point, Baldwin writes that the way toward redemption is through the South *not* the North. While the North believes itself to be superior to the “backwards” ways of the South, it did so as a means of scapegoating the nation to evade its own complicity in white supremacy.

In 1957, Baldwin arrives to Little Rock, Arkansas and is confronted with his lack of familiarity with Southern social mores and taboos. He was, after all, a city boy and while growing up in Harlem was not easy, it was distinct from life in Little Rock. He initially becomes overwhelmed with dread and concern for Black people there. More specifically, he fears for the lives of the children who were sent to integrate the school in this city. He fears for their safety, for their humanity, and the *in*humanity that white Southerners portrayed because of their hatred for Black Americans.

While on his assignment, Baldwin conducts observations of the racialized interaction order that narrates Southern social life. He enters a local restaurant through the front door and is immediately confronted with unwritten social cues suggesting he erred in his entrance. He writes, “every white face turned to stone: the arrival of the messenger of death could not have had a more devastating effect than the appearance in the restaurant doorway of a small, unarmed, utterly astounded black man” (Baldwin, 1972). The waitress, and a white man passing by on the street, remind him of his place: “right around there, boy. Right around there.” Baldwin is fearful and angry and recognizes that his life could very well be at stake for daring to enter the restaurant as another citizen would. More importantly, though, he realizes that, through the menaced glares, coolness, and shock from the white people, these two people believed that they were doing him a favor by reminding him of his place. This social repositioning surprises him, as Baldwin expected that these people would express pure resentment. As he notes (Baldwin, 1972): “[that man] was, indeed, being as kind as can be expected from a guide in hell.”

Much like Baldwin's time in France, his reflections reveal that although he shared similar experiences of racialization with Black folks living in the South, he was unfamiliar with the plight of Black people in the South. That being in a place and of a place was also true internally within the United States. At this realization, Baldwin writes that his, “role was to do a story and avoid becoming one” (Baldwin, 1972). It is easy to assume that he is arguing that he should be a detached, neutral observer and not interfere with the site he is investigating. To the contrary: this quote illustrates that he is acknowledging his status as a *within-nation outsider*, but this status position does not preclude the possibility of making sense of Southern Black life. We know this because he does not represent Black Southern life from the perspective of white Southerners. He finds alternative ways of being that exist outside of the imaginations of white supremacists.

Despite the weight of Jim Crow segregation in the Deep South, Baldwin locates a vibrancy in Black Southern social life. Here is where his position as the Black witness is advantageous. He observes that the hotel where he is staying is also a gathering place for the Black community. It is a place where self-delusion comes to die– where the stoic Baptist preacher can sit across from “the town's loose and fallen ladies and their unstarched men” (Baldwin, 1972). This point about delusion is important: a key theme throughout Baldwin's writing is the prevalence of the dishonesty that Americans continue to believe about themselves. The dishonesty about how they arrived here, how the country emerged within the world, and even what drives people to behave in the way(s) they do. Racism, in his view, is a project rooted in delusion that is accompanied by structural support for those lies. The South, Baldwin argues, is a place where one– Black and white– cannot hide behind that delusion. It is in your face, unlike Northerners who believe they are superior to the ways of the South. The point of the duality of Baldwin's observations in the South– the weight of white supremacy on the one side, and the freedom and joy that Black people experience with each other on the other– demonstrates the Du Boisian observation of the veil (Du Bois, 1903). Black folks in the South have a second sight to not only see white people as they are, but to also see *each other* fully.

The final observation he details in this section is the social and emotional costs of nonviolent protest behavior in the South. He describes two pastors, and one of whom is a grocer. He marvels at the hypocrisy of the American democratic myth: that harmony and unity are at the center of this project. If this was the case, then Reverend D. (the grocer) would not need to arm himself and his children while they watch over his grocery store at night, or the Reverend S. would not have bullet holes riddled in the basement of his church. Both men were passionate about their religious convictions and committed to registering Black voters, which, of course, brought violence to the front steps of their homes and churches. Baldwin expresses sadness as he speaks about the frankness of nonviolence. Today, we have the fortune of merely recognizing (and in some cases memorializing) the nonviolent approach. Baldwin's observations are visceral– he writes (Baldwin, 1972) that his observations of Reverend D. made “the concept of nonviolence real to me” and that the concept of nonviolence “entered the realm of individual and above all private choice and I saw for the first time, how difficult a choice it could be.”

I want to pause here because this point is relevant. The observation of nonviolence should not be viewed within a vacuum. I do not wish for us to conclude that nonviolence is a “natural” response to white supremacist violence or ignore the deeply sacrificial aspects of choosing nonviolence in the face of unrelenting evil that wishes to exterminate your life. Baldwin's framing of this behavior is from a position of deep interpersonal intimacy. Therefore, as he draws near toward this community and its various leaders, his words should move us toward a position of reverence for these people, not pity or truncated, hollow “respect.” Baldwin cautions the reader to not think of nonviolence as a simple decision. Nonviolence is a costly commitment. The loss of property, status, and employment affects an entire family or community system, not just individual community members.

While the *observation* of nonviolence, and the various backstage moments that lead up to the decision to organize for the right to vote happens on the interpersonal level, the connection to the larger pattern of macro-level discrimination should not be lost. It is fitting that Baldwin finishes the first half of the book in the South because, as other scholars of the South detail (Laymon, 2018; Foster, 2020; Wright, 2020): as the South goes, so does the country. The South is not another society that is removed from the North, Midwest, and the Western United States. It is just as much a part of the country as the city that Baldwin claims. Indeed, he understands that “the spirit of the South is the spirit of America” (Baldwin, 1972). To reject and dismiss the South is to reject the genius that built the nation.

In the next section, I turn my attention to the lessons that Baldwin provides in the second half of the book. In this section, he teaches the readers about witnessing the pangs of mass incarceration and its deleterious effects on Black people, their lives, and the Black radical tradition from the position of the within-community outsider.

## Lesson 3: representing the wretched via the within-community outsider

To exist as a witness from the position of the within-community outsider means accepting that even within your own community, you might face ostracization. It is a status position that might result in loneliness. Secondly, this position in the field opens new pathways for the ethnographer to consider “the shape of the wrath to come” (Baldwin, 1972). Baldwin, borrowing from the Christian tradition, deploys this language to speak of the final judgment. This is a striking departure from the liberal optimism in his earlier work, especially in *Fire*. Baldwin's apocalyptic “turn” reflects dissolution– indeed, condemnation– with the American experiment that runs on the wheels of (racial) capitalism (Robinson, 1983). Rather than trusting the individual agency of select people, he turns to an international framework (which he then uses to make sense of his home country) within which individual agency is swallowed up and co-opted by greed and capitalism. Baldwin observes that, without economic justice, the legal gains of the civil rights movement were toothless for many– if not all– Black Americans. He notes that the civil rights had been “rendered moribund” (Baldwin, 1972). Baldwin is not the only Black writer who is thinking about these ideas, though. Other Black writers and artists such as Toni Cade Bambara, Amiri Baraka, Audre Lorde, Sun Ra, and Malcolm X also inhabited an apocalyptic frame to criticize the racial inequality built into America's commitment to capitalism.

Genuine solidarity with the Black radicalism of the late 1960s replaces the liberal optimism he expressed in his earlier writing, and this change is likely a consequence of his remarkable engagement with the Black Panthers. Although he sees the co-opting gimmicks of landlords, jobs, corporations, and politicians in most of the activity in or directed toward the Black ghetto, he finds the same sort of community in the Panthers that he found in the parties in the South and the banlieues in France.

What does this shift in belief in the country mean for political action? Baldwin poses a dilemma: “If [the excluded] attempt to work out their salvation—their autonomy—on terms dictated by those who have excluded them, they are in a delicate and dangerous position, and if they refuse, they are in a desperate one: it is hard to know which case is worse” (Baldwin, 1972). Baldwin suggests that both reform (working within the “terms dictated” by those in power i.e., the pursuit of civil rights) and revolution (“if they refuse,” i.e., separatism, Black Power) are too risky (“delicate and dangerous” or “desperate”). This observation marks him as a within-community outsider. Because he was friends with Malcolm X and befriended members of the Black Panther Party, some civil rights activists did not want to associate with him. Conversely, some members of the Black radical tradition did not like him because he also humanized the civil rights struggle and associated with King, which they believed marked him as a sympathizer with reformist solutions. Although his adjectives are vague in this passage, he *is* clear that the excluded have little freedom to practice their agency and achieve autonomy. He acknowledges that the brunt of capitalism forces the most excluded people to seek alternative ways to reclaim their autonomy.

What does that mean for change, then? He answers this question from two perspectives. From the perspective of the powerless Baldwin writes, “for power to truly feel itself menaced, it must somehow sense itself in the presence of another power—or, more accurately, an energy—which it has not known how to deny and therefore does not really know how to control” (Baldwin, 1972). He elaborates what this cryptic “energy” that power can neither deny nor control might be, and how to acquire it. We must attend to “the people who are the most spectacular recipients of the benefits of this prosperity [that costs millions their lives]” (Baldwin, 1972). In this view, I argue, he is acknowledging that organizations like the Panthers are an exemplar of that counterforce to racial capitalism.

From the perspective of the well-off, however, he notes that eradicating capitalism would cost them too much. He writes that the well-off, “cannot, or dare not, assess or imagine the price paid by their victims, or subjects, for this way of life, and so they cannot afford to know why their victims are revolting. They are forced, then, to the conclusion that the victims—the barbarians—are revolting against all established civilized values” (Baldwin, 1972). As a result, these people “desperately seek out representatives who are prepared to make up in cruelty what both they and the people lack in conviction” (408). Because the well-off refuse to understand the suffering that motivates people to riot, they respond by distancing themselves from “the barbarians” and electing politicians who vow to crack down on the black underclass, such as Richard Nixon, who campaigned, in 1968, on the promise of “law and order.”

Inhabiting the role of the witness allows him the space to align with and critique the responses to oppression and marginalization. He *does* take a position in his assessment of the Black radical tradition, civil rights, and white supremacist counter-behavior. Similar to other sociologists who conduct ethnographies of capitalism and how it manifests in schools, the racial politics of desire, and the criminal justice system (Hoang, 2015; Clair, 2020; Drake, 2022). Baldwin does the work of analysis but he is not so much concerned about proposing an easy solution. Notice that he does not propose new policies or suggest individual reform toward the racialized social system. Another energy entirely– that is not beholden to the cruelties of racial capitalism– is needed.

This disavowal and cruelty “is a formula for a nation's or a kingdom's decline” (Hoang, 2015; Clair, 2020; Drake, 2022). The disavowal prevents the powerful from being able to deny the energy manifested in riots. The election of politicians like Nixon only intensifies this undeniable and uncontrollable energy. In the end, “the victor,” by which Baldwin means the United States, will “become the prisoner of the people he thought to cow, chain, or murder into submission” (Baldwin, 1972). Here is why the apocalyptic turn as a within-nation outsider is useful for ethnographers. He admits that there are hosts of people who are unwilling to change, too, who *enjoy* experiencing the fruits of exploitation at the expense of their fellow countrymen. This is a routine that cannot last for long, in his eyes, and is a formula for the decline of an empire. The excluded must begin “to forge a new morality, to create the principles on which a new world will be built” (Baldwin, 1972). This is what the Panthers and civil rights activists were trying to do with their mobilization, and this is why he turns to apocalyptic language to solve the problem of individual agency. If racial capitalism was the problem, nothing short of its undoing would do. This is a task that could not be solved by changing individual dispositions. Therefore, Baldwin calls for people to wait, endure, and plan for the radically new world that is inexorably coming.

## Conclusion: can anyone be a witness?

Throughout the iterations of the paper, my colleagues (and reviewers) asked me multiple versions of this question: given these terms, can anyone be a witness? The answer is not just a simple “yes.” I am reminded of Toni Cade Bambara's charge: what are [you] pretending not to know today? One cannot be a witness and still hold fast to the seductive lies of American progress. By this, I mean that if we continue to believe that the United States has turned a corner toward some ideal version of progress, we are falling prey to the same delusions that Baldwin wrote about just over fifty years ago. For Baldwin, the sense of responsibility for bearing witness was driven by his recognition of, and refusal to suppress, the fundamental tension emerging, as Leeming (2015, p. 304) puts it, from “beings within himself– the child dancing through life, the intelligence outraged at the nature of things, the madman often blowing the house apart.” *No Name* is a testament to the indivisibility of the personal and the political (and, for our purposes, the distinction between a researcher and a human), as the 1960s snowballed into the unknown futurity of the 1970s. The words shared here still resonate for our moment today.

One must be willing to accept that doing good science does not require a distancing between your identity as a researcher and your identity as an agent. Importantly, one (and one's peers in the scientific community) must accept that refusing to do this distancing does not mean that one's work is “non-scientific” so that one can be a participant in building freedom for all people. This includes being knowledgeable about one's positionality in the field, carefully considering how one interacts with the members of various communities while in the field, refusing to homogenize those members, and to consider the research questions and topics of interest. Importantly, this means having the intellectual humility to defer to marginalized scholars, and members outside of the academic community, as experts and knowledge producers, not as an aside to mainstream scientists. If you accept these terms, then one can operate as a witness.

Additionally, we cannot do effective ethnographic work and ignore the significance of race, gender, class, and sexuality as continuous and stable forces of the social world. These systems of domination are not just variables within our models or details that distort our ethnographic observations– these systems are part of the landscape of inquiry and should be treated as fundamental parts of everyone's lives. Even if a study is not explicitly about race, gender, or class, those elements are there. One cannot be a witness and remain “strangely hesitant” about if these social facts continue to affect our life outcomes and how these social facts are upheld by structures on the macro, meso, and micro-levels (Du Bois, 2000). One's citation practices should reflect this understanding, for instance.

Another question I was asked multiple times is, is being an outsider necessary to be a witness? The multiple layers of “outsideness” as articulated in *No Name* is instructive for ethnographers to keep record of their position(s) in the field and how those positions allow us ways to see—and importantly— not see. Even Baldwin did not cover the full scope of interactions in these different contexts. Even within this provocative book there is terrain that he did not cover. The charismatic, grassroots leadership of Black women was central to the success of the civil rights movement, but was not a point of emphasis in the text. While Baldwin mentions leaders like Martin Luther King, Jr., Malcolm X, and Fred Shuttlesworth, he fails to mention Fanie Lou Hamer or Angela Davis. Baldwin still upheld the responsibilities of witnessing, but he was limited in his insights. There were limitations in the execution of this work. Being an outsider to a community is not a requirement to do the work of witnessing. However, seeing the world from a position of marginality (epistemologically, demographically, and socially) *does* help with doing the work of witnessing because it uncovers what mainstream accounts of inequality often take for granted or ignore as a social fact. The contributions from Black feminists are noteworthy because they cannot afford to ignore race, gender, and sexuality as social forces in their lives. The work of scholars such as Zora Neale Hurston, Nikki Jones, Kemi Adeyemi, Karida Brown, and Mary Pattillo provide rich ethnographic insights that provide answers to questions that are too often neglected by mainstream sociologists. Instead of pursuing generalizability, the work of these scholars reminds us that what we call the “marco” cannot exist without individuals and the communities that they inhabit. The inequality that we regularly see is not “natural” but requires people to buy into it and believe it, which also means that we have the capacity and obligation to disrupt those systems as well.

Finally, I will return to the apocalyptic future point that I shared in the paper's abstract and discuss how Baldwin's lessons for witnessing matter for practitioners and ethnographers. We must forego the liberal delusion that the nation has encountered significant progress. Perhaps it might be best to dismiss the term progress altogether, especially if it is used in vacuous terms. As Spillers (2022) reminds us, whenever there is a “first” of something (e.g., first Black president or vice-president), that means it has arrived too late. This is not to say that *No Name* is devoid of hope, though. Baldwin does express hope, but differently from the “reasonable Black and whites” that he mentioned in *Fire*. In *No Name*, he expresses hope in the possibilities of the revolutionary forces that are fueling movements within the Black radical tradition. This is not the same as pursuing specific policy goals that the civil rights movement tried to accomplish. Instead, hope lies in the recognition of the Black radical tradition's potential for rupturing current social arrangements that are grounded within the teleological myths of American racial progress.

The inspiration for the title of the text comes from the Book of Job. Baldwin uses the passage from which it comes to articulate the fate of the wicked. The passage prophesies the annihilation of any memory of wicked people, which I believe he meant as a layered warning to white Americans and the global racial order. The threat of the fire next time– as something that would come if interracial coalitions were not formed– did not hit hard enough. Ten years after its release, Baldwin returns to us with yet another warning, and refusing to put hope in his white countrymen. The same year that this book was published, Richard Nixon's “law and order” approach devastated and disrupted the lives of millions of Black Americans. The conditions for the Black underclass are worse than they were in the 1970s. Mass incarceration continues to solve the problem that Baldwin observes: “that this country does not know what to do with its black population now that the blacks are no longer a source of wealth” (432). And with the emergence of movements like the Movement for Black Lives in response to the “plunder” of Black lives (Coates, 2015), we are still witnessing how white Americans continue to elect “representatives who are prepared to make up in cruelty what both they and the people lack in conviction” (432). If we take a look at the expulsion of two Democratic State Representatives in Tennessee, the rise of moral panic about critical race theory and the subsequent legislation in Florida to make it illegal to teach accurate U.S. history, and attempts by judges to close libraries that house banned books, it is clear that we need to continue to take up the challenge of witnessing. The work of cultural criticism must continue in order to participate in the ongoing pursuit of freedom.

## Data availability statement

The original contributions presented in the study are included in the article/supplementary material, further inquiries can be directed to the corresponding author.

## Author contributions

The author confirms being the sole contributor of this work and has approved it for publication.

## Footnotes

^1^See “A Suggestion on the Negro Problem” by Charlotte Perkins Gilman, published in the *American Journal of Sociology*. The (1908) essay begins with the assertion that the “superior” race must find a practical means for speeding up the “racial evolution” of that “large body of aliens, of a race widely dissimilar and in many respects inferior, whose present status is to us a social injury.” Gilman argues that people of African descent were largely incapable of progressing to the level of whites. The “problem,” she noted, was that, “He [the Black person] is here; we can't get rid of him; it is all our fault; he does not suit us as he is; what can we do to improve him?” Astoundingly, she suggests that each state enlist all “negroes below a certain grade of citizenship” into a quasi-military organization that would perform dignified labor for society and thereby develop the work habits and personal discipline that will make them “productive” members of society. This is the type of person that would likely refute the historical account that Du Bois provides in his magisterial work.

^2^I am also reminded of Zora Neale Hurston's research strategy in *Barracoon*. Hurston did not elect to interview former slave masters to understand the violent history that Black Americans endured. She understood that there was a concerted effort to silence Black people from telling their own stories and instead replace them with watered down and false narratives about benevolent slave masters. Yet, according to today's publishing standards in sociology, most reviewers would likely argue that her study design is weak or invalid because of her proximity to the subject matter and the size of her research sample (*n* = 1). Despite these ludicrous criticisms, Hurston inhabited the role of the witness; she understood that to understand the true scope of this vile institution required speaking to people who survived the violence of the Middle Passage and slavery firsthand. This testimonial text helps us see the critical and historical singularity of slavery's violence, thereby connecting the past, present, and future simultaneously through Koussula's narrative.

^3^See: Baldwin (1962). “The Artist's Struggle for Integrity.”
